# Dissecting the Immunological Profiles in *NSD3*-Amplified LUSC through Integrative Multi-Scale Analyses

**DOI:** 10.3390/cancers14204997

**Published:** 2022-10-12

**Authors:** Duo Xu, Shengchen Liu, Xi Wu, Thomas M. Marti, Patrick Dorn, Ralph A. Schmid, Ren-Wang Peng, Yongqian Shu

**Affiliations:** 1 Department of Oncology, The First Affiliated Hospital of Nanjing Medical University, Nanjing 210029, China; 2 Department of Cardio-Thoracic Surgery, Nanjing First Hospital, Nanjing Medical University, Nanjing 210006, China; 3 Division of General Thoracic Surgery, Inselspital, Bern University Hospital, University of Bern, 3008 Bern, Switzerland; 4 Department for BioMedical Research (DBMR), Inselspital, Bern University Hospital, University of Bern, 3008 Bern, Switzerland

**Keywords:** lung squamous cell carcinoma (LUSC), *NSD3*, tumor immune microenvironment (TIME), unfolded protein response (UPR), immunotherapy

## Abstract

**Simple Summary:**

Although cancer immunotherapy has become a “game changer” in treating LUSC patients, challenges still prevail due to the heterogeneous response and insufficient predictive biomarkers. Recently, *NSD3*, a neighbor gene of *FGFR1*, was identified as a key genetic driver of LUSC tumorigenesis. While previous studies have reported the relevance of *NSD3* in innate antiviral immunity, the association of *NSD3* with the TIME remains an open question requiring further investigation. We first show that *NSD3* gene amplification is associated with an immune-desert phenotype and correlated with a worse immunotherapy outcome. Further molecular characterizations pinpoint that the high activity of UPR signaling might be a pivotal mediator for the non-inflamed TME phenotype of *NSD3*-amplified LUSC. Concordantly, the pharmaco-transcriptomic correlation analysis indicated that the intervention of UPR signaling could be a promising synthetic lethality target for *NSD3*-amplified LUSC. Our findings reveal a previously unappreciated immunological role for *NSD3* in LUSC pathobiology and provide therapeutic rationales for this daunting disease.

**Abstract:**

The histone H3 lysine 36 (H3K36) methyltransferase *NSD3*, a neighboring gene of *FGFR1*, has been identified as a critical genetic driver of lung squamous cell carcinoma (LUSC). However, the molecular characteristics, especially the immunological roles of *NSD3* in driving carcinogenesis, are poorly understood. In this study, we systematically integrated multi-omics data (e.g., genome, transcriptome, proteome, and TMA array) to dissect the immunological profiles in *NSD3*-amplified LUSC. Next, pharmaco-transcriptomic correlation analysis was implemented to identify the molecular underpinnings and therapeutic vulnerabilities in LUSC. We revealed that *NSD3*-amplified LUSC presents a non-inflamed tumor immune microenvironment (TIME) state in multiple independent LUSC patient cohorts. Predictably, elevated *NSD3* expression was correlated with a worse immunotherapy outcome. Further molecular characterizations revealed that the high activity of unfolded protein response (UPR) signaling might be a pivotal mediator for the non-immunogenic phenotype of *NSD3*-amplified LUSC. Concordantly, we showed that *NSD3*-amplified LUSCs exhibited a more sensitive phenotype to compounds targeting UPR branches than the wild-type group. In brief, our multi-level analyses point to a previously unappreciated immunological role for *NSD3* and provide therapeutic rationales for *NSD3*-amplified squamous lung cancer.

## 1. Introduction

Lung squamous cell carcinoma (LUSC), a primary subtype of non-small cell lung cancer, is one of the leading causes of cancer-related mortality worldwide, with an abysmal prognosis and aggressive phenotype [1,2]. Although many efforts have been made to investigate the molecular underpinnings and actionable driver mutations of LUSC tumorigenesis, the therapeutic landscapes of LUSC have hardly changed for decades, with no targeted therapy available [3,4,5].

Comprehensive genomic studies have revealed that approximately 20% of LUSC patients have amplification of the chromosomal region of 8p11–12, among which *FGFR1* has been identified as the leading candidate driver within this region for a long time [6,7]. However, clinical trials with FGFR-TKIs for LUSC patients harboring *FGFR1* alterations are unsuccessful, raising the possibility that LUSC might have other potential driver mutations that confer the aggressive nature of tumor growth [8,9]. Recently, the histone H3 lysine 36 (H3K36) methyltransferase *NSD3*, a neighboring gene of *FGFR1*, has been defined as a critical mutational driver of LUSC tumorigenesis [10]. Beyond LUSC, the oncogenic activity of *NSD3* has also been characterized in breast cancer and other malignancies [11]. However, the molecular underpinnings of *NSD3* gene amplification in driving carcinogenesis are still poorly understood.

Although immunotherapy with immune checkpoint blockade (ICB) has achieved remarkable clinical efficacy for NSCLC, challenges still prevail due to the heterogeneous response [2]. As a complex entity involving various immune components, the tumor immune microenvironment (TIME) considerably impacts the response and acquired resistance to ICBs [12,13,14]. Of note, mounting evidence has demonstrated apparent heterogeneity in the tumor immunogenicity of LUSC patients, further highlighting that biomarker-driven stratification for immunotherapy is urgently needed [15,16]. Previous studies have reported the relevance of *NSD3* in innate antiviral immunity [17], but the association of *NSD3* with the TIME remains an open question.

By utilizing multi-scale analyses, we have provided the first evidence that *NSD3*-amplified LUSC shapes a non-immunogenic phenotype and consequently correlates with worse clinical outcomes of ICBs. Further molecular characterizations revealed that unfolded protein response (UPR) signaling might be a pivotal mediator for such a non-inflamed TIME phenotype. More importantly, a pharmaco-transcriptomic correlation analysis indicated that the intervention of UPR signaling could be an actionable synthetic lethality target for *NSD3*-amplified LUSC. These findings point to a previously unappreciated immunological role for *NSD3* and provide therapeutic rationales for treating this devasting disease.

## 2. Materials and Methods

### 2.1. Data Retrieval and Preprocessing

The RNA sequencing (RNA-seq) data (FPKM value), copy number variants (CNVs), genomic mutation data, and clinical-related information of The Cancer Genome Atlas (TCGA) LUSC dataset were obtained from the online data portal UCSC Xena (https://xenabrowser.net/datapages/, accessed on 1 March 2022). Data on RNA-seq were log2 transformed, and the GISTIC algorithm processed the CNV information. In addition, the CPTAC LUSC cohort downloaded from the cBioPortal (http://cbioportal.org, accessed on 1 March 2022) was included in our study [18]. Moreover, transcriptomic data from transgenic mice carrying a lung-specific active mutant (T1232A) or genetic knockout of *Nsd3* (GSE149212 & GSE149482) and one immunotherapy-related cohort GSE135222(NSCLC) were downloaded from the Gene Expression Omnibus (GEO) [10,19]. CRISPR-knockout gene scores of *NSD3*, *EIF4EBP1,* and *LSM1* in NSCLC cell lines were downloaded from the Cancer Dependency Map Data Portal (DepMap_22Q2_Public + Score_Chronos) [20]. Drug sensitivity datasets were downloaded from the Genomics of Drug Sensitivity in Cancer (GDSC) [21].

### 2.2. Identification of Differentially Expressed Genes (DEGs) and Functional Enrichment Analysis

Patients were classified according to *NSD3* genetic status. The empirical Bayesian approach of the limma R package was applied to identify DEGs from the gene expression profile between groups (amplification vs. non-amplification). The criteria for determining differential DEGs were set with an adjusted *p* value < 0.05. We determined the common DEGs using the software VennDiagram R package. Gene Ontology (G.O.) and Kyoto Encyclopedia of Genes and Genomes (KEGG) enrichment analyses were performed using the ClusterProfiler R package.

### 2.3. Estimation of the Immunological Profiles of the TME in LUSC

The ESTIMATE algorithm, a method that uses gene expression signatures to infer the fraction of stromal and immune cells in tumor samples, was applied to assess the stromal score, immune score, ESTIMATE score, and tumor purity [22]. Information on 122 immunomodulators (MHC, immune receptors, chemokines, and immune stimulators) and 33 well-known effector genes of tumor-infiltrating immune cells (TIICs) were collected from previous studies [23,24]. In addition, to avoid the errors caused by various algorithms when estimating the levels of TIICs, five independent algorithms: TIMER, EPIC, MCP-counter, quanTIseq, and TISIDB, were applied to comprehensively evaluate the relative abundances of TIICs [25,26,27,28,29]. We also identified gene signatures of 29 immune cell types and immune-related pathways from a previous study [30]. Exhausted- and cytotoxic T-cell signatures were independently scored as the sum of the corresponding gene sets (exhausted: *TIGIT, HAVCR2, CTLA4, LAG3,* and *PDCD1*; cytotoxic: *GZMA, GZMB, GZMK, IFNG,* and *IL2*).

Furthermore, a single-sample gene set enrichment analysis (ssGSEA) was performed to evaluate the activities of the cancer immunity cycle according to the expression levels of specific signatures. Seven major steps are comprised as follows: step 1, the release of cancer cell antigens; step 2, cancer antigen presentation; step 3, priming and activation; step 4, trafficking of immune cells to tumors; step 5, infiltration of immune cells into tumors; step 6, recognition of cancer cells by T-cells; and step 7, killing of cancer cells [24,31]. After that, 22 inhibitory immune checkpoints with therapeutic potential, 18 genes associated with T-cell inflammation as the T-cell inflamed score, and the immunophenoscore (IPS) were collected to predict the clinical response of ICBs [23,32,33]. To dissect the role of *NSD3* gene amplification in modulating cancer immunity, we evaluated the immunological pattern of the TME concerning the above aspects.

### 2.4. Ethics Statements

The LUSC tissue microarray (TMA, HLugS180Su01), which contains 90 LUSC tumor tissue and adjacent normal samples, was obtained from Outdo Biotech (Shanghai, China). The HBlaU079Su01 microarray ethical approval for the study of tissue microarray slides was granted by the Clinical Research Ethics Committee, Outdo Biotech (SHYJS-CP-1810010, 2018.10; Shanghai, China).

### 2.5. Immunohistochemistry of LUSC Tissue Microarray

Immunohistochemistry (IHC) staining was directly conducted on the HLungS180Su01 TMA with standard procedures. The primary antibodies used were as follows: anti-*NSD3* (1:1000 dilution, Cat.11345-1-AP, Proteintech, Wuhan, China), anti-PD-L1 (Ready-to-use, Cat. GT2280, GeneTech, Shanghai, China), and anti-CD8 (Ready-to-use, Cat. PA067, Abcarta, Suzhou, China). Antibody staining was visualized with DAB and hematoxylin counterstain, and whole slide images were scanned using Aperio Digital Pathology Slide Scanners.

### 2.6. Semiquantitative Scoring

Two pathologists independently assessed all stained slides. The percentage of *NSD3*- and PD-L1-positive cells in the whole slide region was scored as 0–4: 0 (<1%), 1 (1–5%), 2 (6–25%), 3 (26–50%) and 4 (>50%). Moreover, the staining intensity was scored as 0–3: 0 (negative), 1 (weak), 2 (moderate), and 3 (strong). After that, the percentage of positive cells multiplied by the staining intensity was calculated as the immunoreactivity score (IRS). The infiltration level of CD8+ T-cells was assessed by estimating the percentage of cells with strong membrane staining intensity.

### 2.7. Classification of Immune Subtypes

According to the spatial distribution of CD8+ T-cells, tumors were divided into three major phenotypes, as previously described [24]. The “inflamed phenotype” included CD8+ T cells located in the tumor parenchyma; the “excluded phenotype” included CD8+ T cells located in the stroma surrounding the tumor but not in the parenchyma, and the “deserted phenotype” was characterized by the absence of CD8+ T cells in both the tumor parenchyma and stroma. The non-inflamed immune state can be defined by either excluded or deserted phenotypes.

### 2.8. Prediction of Clinical Drug Response

The R package “pRRophetic” was used to predict each patient’s response to common clinical chemotherapeutic drugs [34]. The half-maximal inhibitory concentration (IC50) of each sample was calculated by ridge regression. In addition, a 10-fold cross-validation based on the CGP training set was applied to evaluate the prediction accuracy. Here, default options were used for all parameters.

### 2.9. Statistical Analysis

Comparisons between two groups were conducted using Student’s *t*-test or Wilcoxon rank-sum test according to the normality. One-way or two-way analysis of variance (ANOVA) was performed to determine the comparisons among more than two groups. Tumor samples within all datasets were divided into two groups based on the best-separation cut-off value to plot the Kaplan–Meier survival curves using the “survminer” and “survival” R packages. Predictive values of categorical variables were assessed using the log-rank test. GraphPad Prism 8 or R software (version 4.0.4, http://www.rproject.org, accessed on 1 May 2022) was utilized for statistics, and a *p*-value < 0.05 was considered statistically significant.

## 3. Results

### 3.1. NSD3 Is a Critical Genetic Driver of LUSC Tumorigenesis

**Figure 1:**
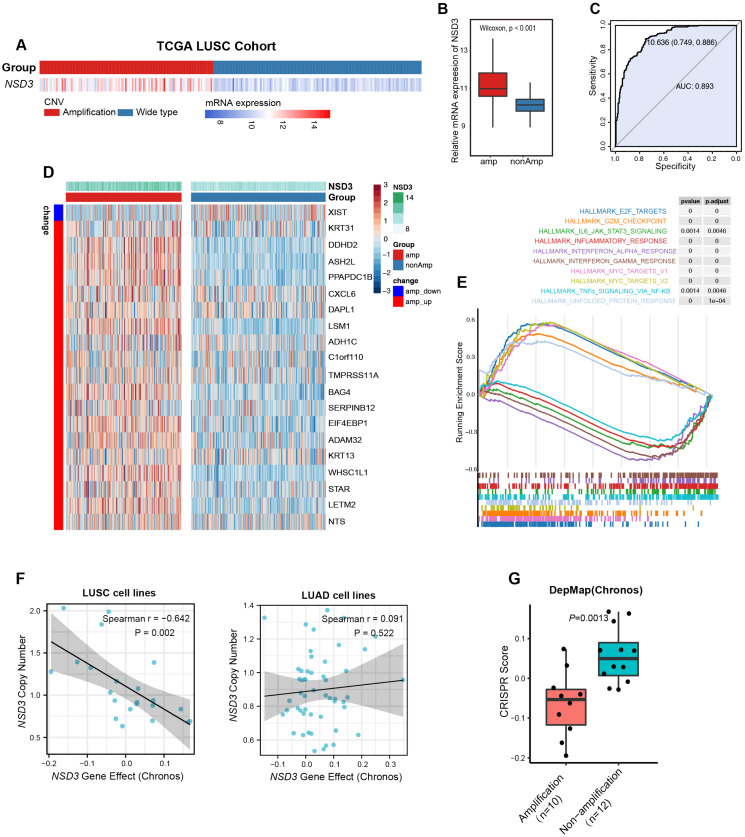
*NSD3* is a key mutational driver of LUSC carcinogenesis. (**A**,**B**) Correlation analysis between the amplification status and mRNA expression of *NSD3* in the TCGA LUSC patient cohort (*n* = 491). The Wilcoxon rank-sum test was used for comparison, and *p* < 0.05 was considered significant. (**C**) Receiver operating characteristic (ROC) curves manifest that for *NSD3*, the corresponding mRNA level is a sensitive marker to predict its amplification state. (**D**,**E**) The top 20 differentially expressed genes (DEGs) between the non- and amplification groups of *NSD3* shown in the heatmap were ranked by the adjusted *p*-value (**D**). Data were downloaded from the TCGA LUSC cohort (*n* = 491). Gene set enrichment analysis (GSEA) was performed based on the transcriptomic data from the TCGA-LUSC cohort stratified by *NSD3* genetic status. (**F**) Correlation analysis between the copy number of *NSD3* and CRISPR genetic knockout score across LUSC (*n* = 22) and LUAD cell lines (*n* = 52). Data were downloaded from The Cancer Dependency Map Project (DepMap; 22Q2 Chronos). (**G**) The difference in the CRISPR genetic knockout score between the non- and amplification groups of *NSD3* in LUSC cell lines (*n* = 22). Data were downloaded from The Cancer Dependency Map Project (DepMap; 22Q2 Chronos). A two-tailed unpaired *t*-test was used for comparison, and *p* < 0.05 was considered significant.

As one of the more common genetic alterations in LUSC, the *NSD3* amplification state strongly correlates with corresponding mRNA levels (Figure 1A,B). Accordingly, the mRNA expression of *NSD3* is a superior predictor of its amplification state (Figure 1C). Moreover, transcriptomic data from The Cancer Genome Atlas (TCGA) showed that *NSD3*-amplified LUSC patients featured a panel of differentially expressed genes (DEGs), such as *LETM2, STAR, KRT13, ADAM32,* and *EIF4EBP1,* which have considerable impacts on cell growth, metastasis, and anti-tumor immunity in various cancers (Figure 1D, Supplementary Table S1).

Strikingly, Gene Set Enrichment Analysis (GSEA) revealed that *NSD3*-amplified tumors were highly enriched in MYC targets, E2F targets, G2-M checkpoint, and unfolded protein response (UPR) but displayed lower activity in immune-related pathways (e.g., interferon-alpha/gamma response, TNFα-NF-κB signaling, and inflammatory response) (Figure 1E, Supplementary Table S2). In addition, the *NSD3*-amplified group displayed no significant differences in genomic mutations or tumor mutation burden (TMB) (Supplementary Figure S1A–C). Furthermore, genetic knockout of *NSD3* specifically led to a potent anti-tumor effect in *NSD3*-amplified LUSC cell lines (Figure 1F,G, Supplementary Table S3). Together, these data confirm a pivotal oncogenic role for *NSD3* in LUSC tumorigenesis and indicate that *NSD3* might participate in regulating anti-tumor immunity.

### 3.2. NSD3 Shapes a Non-Inflamed TIME in LUSC

**Figure 2:**
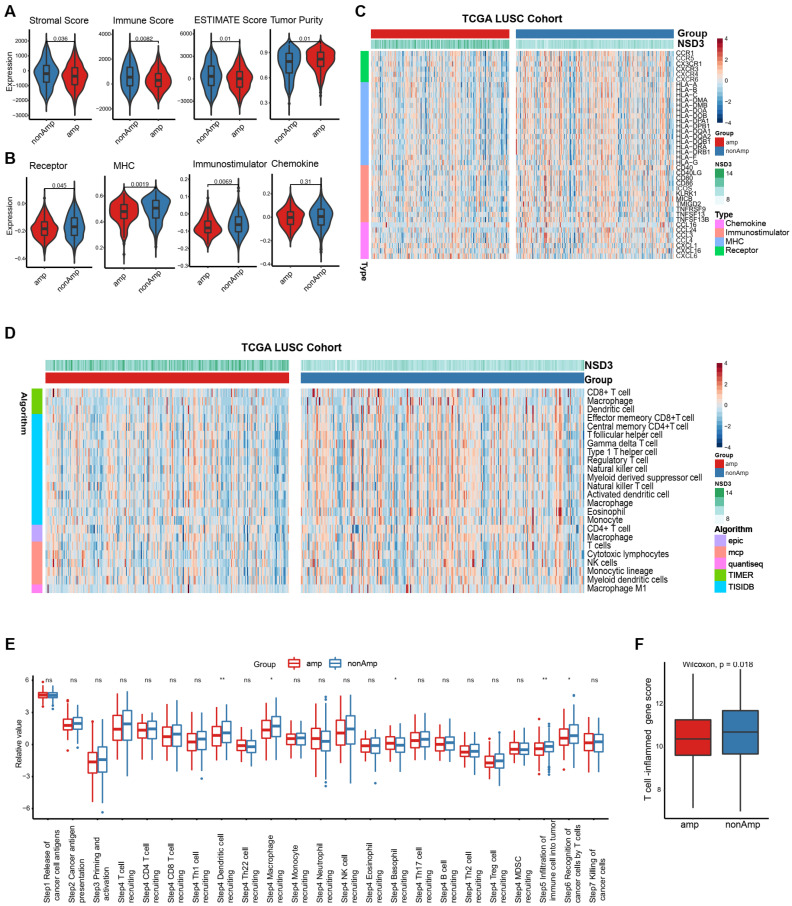
*NSD3* shapes a non-immunogenic phenotype in the TCGA-LUSC. The Wilcoxon rank-sum test was used for all comparison, and *p* < 0.05 was considered significant. (**A**) Distribution plots of tumor purity, ESTIMATE score, immune score, and stromal score were calculated using the ESTIMATE algorithm in the non- and amplification groups of *NSD3* across the TCGA LUSC cohort (*n* = 491). (**B**,**C**) The expression pattern of immunomodulators (chemokines, immunostimulators, MHC, and receptors) in the non- and amplification groups of *NSD3* across the TCGA LUSC cohort (*n* = 491). Data with statistical significance were shown in (**C**). (**D**) The expression levels of tumor-infiltrating immune cells were calculated using five various algorithms (TIMER, EPIC, MCP-counter, quanTIseq, and TISIDB) in the non- and amplification groups of *NSD3* across TCGA LUSC patients’ cohort (*n* = 491). Data with statistical significance were shown. (**E**) The activities of various steps involved in the cancer immunity cycle were calculated by the ssGSEA algorithm in the non- and amplification groups of *NSD3* across the TCGA LUSC cohort (*n* = 491). ns: none significant; * *p* < 0.05; ** *p* < 0.01. (**F**) The difference in the T-cell inflamed gene score between the non- and amplification groups of *NSD3*.

Considering that an altered expression pattern of immune-related pathways was observed in *NSD3*-amplified tumors, we speculated that *NSD3* might play an immunological role in LUSC. To investigate the possible correlation between *NSD3* gene amplification and anti-tumor immunity, we sought to evaluate the immune landscapes within *NSD3*-amplified LUSC samples using a multi-faceted approach. Generally, we found that *NSD3*-amplified tumors exhibited lower stromal, immune, and ESTIMATE scores but a higher tumor purity than the wild-type tumors (Figure 2A). In line with this, *NSD3*-amplified tumors were accompanied by a decreased expression pattern in a majority of immunomodulators (e.g., MHC molecules, immunostimulators, and immune receptors), indicating a weak capacity for antigen presentation and processing as well as the recruitment of effector TIICs into the TME (Figure 2B,C, Supplementary Table S4).

To better characterize the TIME, we further incorporated five independent algorithms (TIMER, EPIC, MCP-counter, quanTIseq, and TISIDB) to assess the infiltration status of immune cells in LUSC. Our data showed that the infiltration levels of most immune cells, particularly the cytotoxic components (e.g., CD8^+^ T-cells, NK cells, and macrophages), were generally downregulated in the *NSD3*-amplified group (Figure 2D, Supplementary Figure S2A,B and Tables S5 and S6). In line with the above findings, we found that *NSD3* gene amplification was characterized by reduced activity of major steps involved in the cancer-immunity cycle, especially in Step 4 trafficking of immune cells to tumors (e.g., dendritic cell, macrophage, and basophil recruiting), Step 5 the infiltration of the immune cell into cancer, and Step 6 recognition of cancer cells by T-cells (Figure 2E. Meanwhile, reduced expression of the T-cell inflamed score, a predictive marker for the immunogenic state [33], was observed in the *NSD3*-amplified group, further strengthening the notion that *NSD3*-amplified LUSC features a non-immunogenic phenotype (Figure 2F).

**Figure 3:**
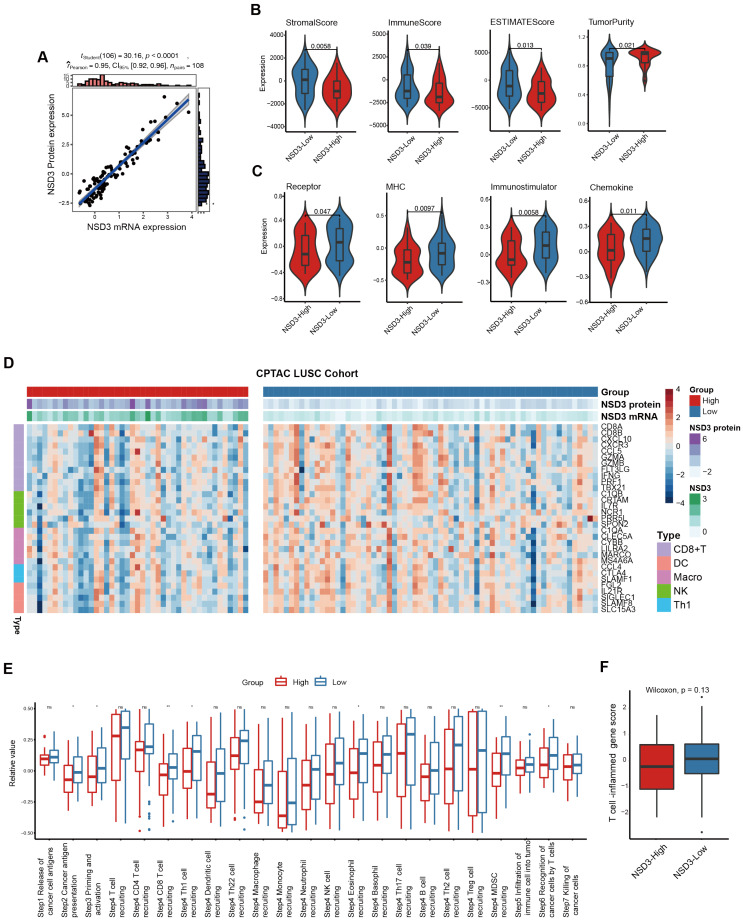
*NSD3* defines a non-immunogenic phenotype in the CPTAC-LUSC. The Wilcoxon rank-sum test was used for comparison, and *p* < 0.05 was considered significant. (**A**) Correlation analysis between the mRNA and protein expression of *NSD3* in the CPTAC LUSC cohort (*n* = 108). (**B**) Distribution plots of tumor purity, ESTIMATE score, immune score, and stromal score were calculated using the ESTIMATE algorithm in the *NSD3*-low and *NSD3*-high groups according to the corresponding protein levels at the cut-off of zero across the CPTAC LUSC cohort (*n* = 108). (**C**) The difference in immunomodulators expression (chemokines, immunostimulators, MHC, and receptors) between the *NSD3*-low and *NSD3*-high groups according to the corresponding protein levels at the cut-off of zero across the CPTAC LUSC patients’ cohort (*n* = 108). (**D**) The expression pattern of immune cell-related gene markers in the *NSD3*-low and *NSD3*-high groups according to the corresponding protein levels at the cut-off of zero across the CPTAC LUSC cohort (*n* = 108). (**E**) The activities of various steps involved in the cancer immunity cycle were calculated by the ssGSEA algorithm in the *NSD3*-low and *NSD3*-high groups according to the corresponding protein levels at the cut-off of zero across the CPTAC LUSC cohort (*n* = 108). ns: none significant; * *p* < 0.05; ** *p* < 0.01. (**F**) The difference in the T-cell inflamed gene score between the *NSD3*-low and -high groups according to the corresponding protein levels at the cut-off of zero in the CPTAC LUSC cohort (*n* = 108).

Subsequently, we incorporated the CPTAC LUSC cohort as an external validation dataset. First, based on the proteome dataset of the CPTAC cohort, we validated that *NSD3* gene expression correlated strongly with its protein levels (Figure 3A). Next, we divided the cohort into *NSD3*- high and low groups according to the corresponding protein levels at the cut-off of zero. As expected, a similar suppressive anti-tumor immunity was observed in LUSC patients with high protein levels of *NSD3* (Figure 3B–F, Supplementary Figure S2C and Tables S7 and S8). In summary, by mining two large independent LUSC patient cohorts (TCGA and CPTAC), we reveal that *NSD3*-amplified LUSC presents a non-inflamed TME state, which might contribute to a poor clinical response to immunotherapy.

### 3.3. The Association between NSD3 and Immune Infiltrates in LUSC

**Figure 4:**
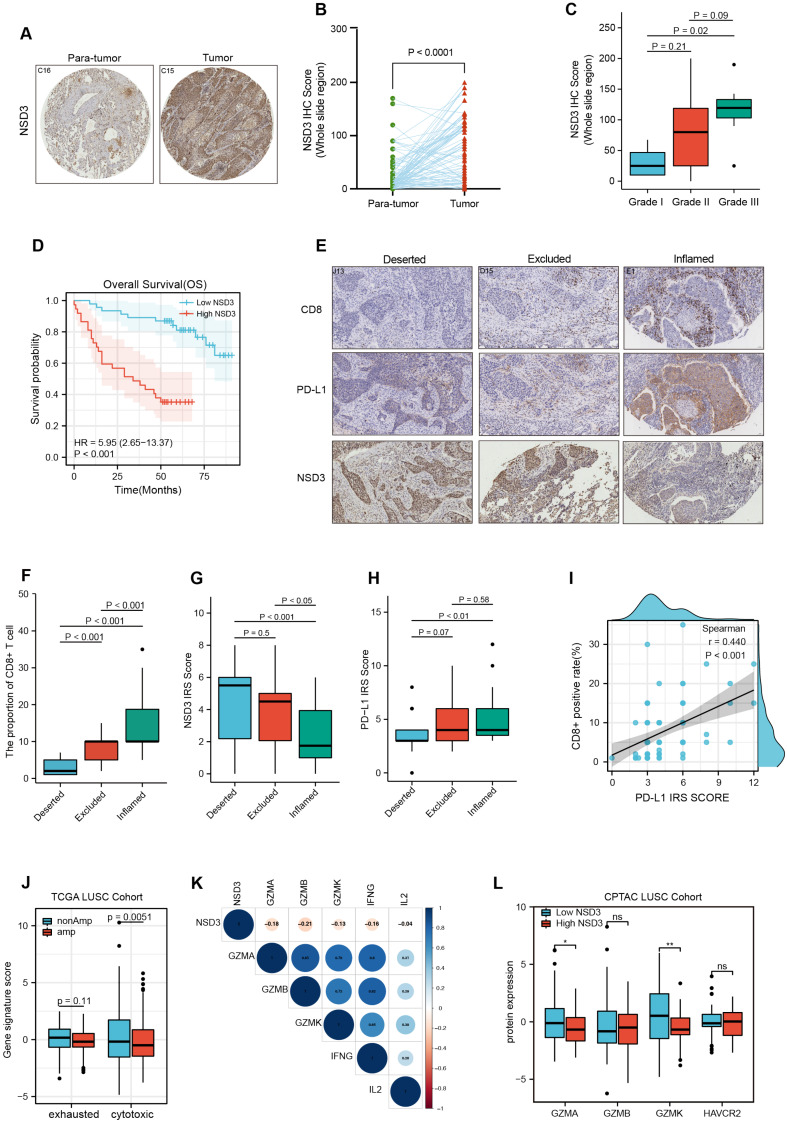
The association between *NSD3* expression and immune infiltrates in the LUSC tissue microarray. In all, *p* < 0.05 was considered significant. (**A**,**B**) The difference in the *NSD3* IHC score (whole slide region) between the para-tumor and tumor groups in a LUSC tissue microarray (*n* = 90; (**B**). Representative IHC images are shown in (**A**). Original overall magnification, ×200. Scale bar: 50 μM. A two-tailed unpaired *t*-test was used for comparison. (**C**) The expression levels of the *NSD3* IHC score (whole slide region) in different pathological stages of LUSC patients (*n* = 90). One-way analysis of variance (ANOVA) followed by Dunnett’s multiple comparison test was used for comparison. (**D**) Kaplan–Meier analysis of LUSC patients based on the protein level of *NSD3* in the TMA array (*n* = 90) with high- (in red) or low- (in blue) were stratified by the optimal cut-off value of the individual protein expression across all patients using the surv_cutpoint function in the R “maxstat” package. Log-rank test was used for comparison. (**E**) IHC staining for indicated proteins in the LUSC TMA array (*n* = 90). Representative IHC images are shown. Original overall magnification, ×200. Scale bar: 50 μM. (**F**–**H**) Infiltration levels of CD8+ T-cells and the expression levels of *NSD3* (**G**) and PD-L1 (**H**) in three different immune subtypes of LUSC samples (*n* = 90). One-way analysis of variance (ANOVA) followed by Dunnett’s multiple comparison test was used for comparison. (**I**) Correlation analysis between the infiltration of CD8+ T-cells and the PD-L1 IRS score in the LUSC TMA array (*n* = 90). (**J**) The difference in the exhausted- and cytotoxic T-cell signature between the non- and amplification groups of *NSD3* in the TCGA LUSC cohort (*n* = 491). Two-way analysis of variance (ANOVA) followed by Sidak’s multiple comparison test was used for comparison. (**K**) Heatmap showing the Spearman correlation coefficients between the mRNA expression of *NSD3* and cytotoxic T-cell-related gene markers in the TCGA LUSC cohort (*n* = 491). (**L**) The difference in the protein levels of cytotoxic T-cell-related gene markers (GZMA, GZMB, and GZMK) and exhausted T-cell gene marker (HAVCR2) between the *NSD3*-low and -high groups in the CPTAC LUSC cohort (*n* = 108). Two-way analysis of variance (ANOVA) followed by Sidak’s multiple comparison test was used for comparison. ns: none significant; * *p* < 0.05; ** *p* < 0.01.

To continually move forward to the clinical settings, our findings were examined in an internal LUSC TMA cohort, which included 90 paired para-tumor and LUSC samples (Supplementary Figure S3A,B). Our data further confirmed that *NSD3* was highly expressed in LUSC patient samples compared to paired normal control samples and associated with aggressive phenotypes and poor prognosis (Figure 4A–D). We next divided the LUSC cohort into three immunophenotypes, deserted, excluded, and inflamed, according to the spatial distribution of CD8+ T-cells (Figure 4E). Notably, *NSD3* exhibited the highest protein expression in the immune-desert phenotype, which displayed an absent CD8+ T-cell infiltration and relatively lower expression of PD-L1 (Figure 4F–I).

To further elucidate the internal links between *NSD3* and CD8+ T cells, we performed data mining of T-cell states in the TCGA-and CPTAC-LUSC cohorts. Interestingly, our results showed that *NSD3*-amplified LUSC was characterized by a downregulated molecular signature of cytotoxic T-cells (Figure 4J). Moreover, *NSD3* displayed a strong negative correlation with the molecules (*GZMA, GZMB, GZMK,* and *IFNG*) from the cytotoxic T lymphocytes (CTLs) (Figure 4K). In line with the above findings, the protein levels of Granzyme A (GZMA) and Granzyme K (GZMK) were significantly decreased in the LUSC patients with a high level of *NSD3*, but no apparent difference in the T-cell exhaustion marker, TIM3 (HAVCR2) (Figure 4L). Overall, the above evidence further supports our speculation that *NSD3* gene amplification defines a non-inflamed TIME; however, how *NSD3* affects the suppressive immune microenvironment, particularly the interplay between *NSD3* and CTLs, remains to be further elucidated.

### 3.4. Immunotherapy Outcome Prediction by NSD3

**Figure 5:**
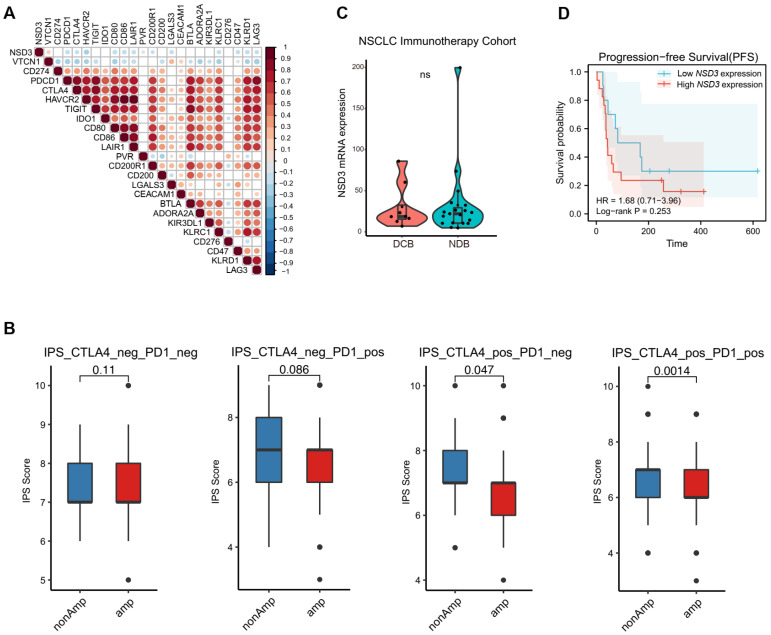
Clinical predictive value of *NSD3* for immunotherapy outcome in LUSC. (**A**) Heatmap showing the Spearman correlation coefficients between the mRNA expression of *NSD3* and a panel of inhibitory immune checkpoints in the TCGA LUSC cohort (*n* = 491). (**B**) Immunophenoscore (IPS) distribution plot in the non- and amplification groups of *NSD3* across the TCGA LUSC cohort (*n* = 491). The Wilcoxon rank-sum test was used for comparison, and *p* < 0.05 was considered significant. (**C**) mRNA expression of *NSD3* in the different clinical response groups (DCB: durable clinical benefit; non-durable clinical benefit) based on the transcriptomic dataset from the NSCLC immunotherapy cohort (*n* = 27; GSE135222). A two-tailed unpaired *t*-test was used for comparison, and *p* < 0.05 was considered significant. ns: none significant. (**D**) Kaplan–Meier analysis based on the mRNA level of *NSD3* in the NSCLC immunotherapy cohort (*n* = 27; GSE135222) with high- (in red) or low- (in blue) expression was stratified by the optimal cut-off value of the individual expression across all patients using the surv_cutpoint function in the R “maxstat” package. The log-rank test was used for comparison, and *p* < 0.05 was considered significant.

As expected, *NSD3* expression was found to be negatively correlated with the majority of inhibitory immune checkpoints (e.g., PD-1, CTLA-4, and CD86), and more importantly, a lower expression of immunophenoscores (IPS) was shown in the *NSD3*-amplified group, suggesting that *NSD3*-amplified LUSC might have a weak response to corresponding ICBs (Figure 5A,B, Supplementary Table S9) [23]. To further investigate the predictive capacity of *NSD3* for the clinical outcome of immunotherapy, we mined bulk RNA-Seq data and clinical information from one NSCLC immunotherapy cohort treated with anti-PD-1/PD-L1. Although there was no noticeable difference between the response state (DRB vs. NRB), elevated *NSD3* expression was tightly correlated with shorter progression-free survival (PFS), indicating that *NSD3* might be a promising predictor of immunotherapy outcome (Figure 5C,D). Future studies are highly encouraged to validate the above observations by evaluating response data from the LUSC immunotherapy cohort.

### 3.5. Internal Links between the Unfolded Protein Response and Non-Immunogenic Feature of NSD3-Amplified LUSC

**Figure 6:**
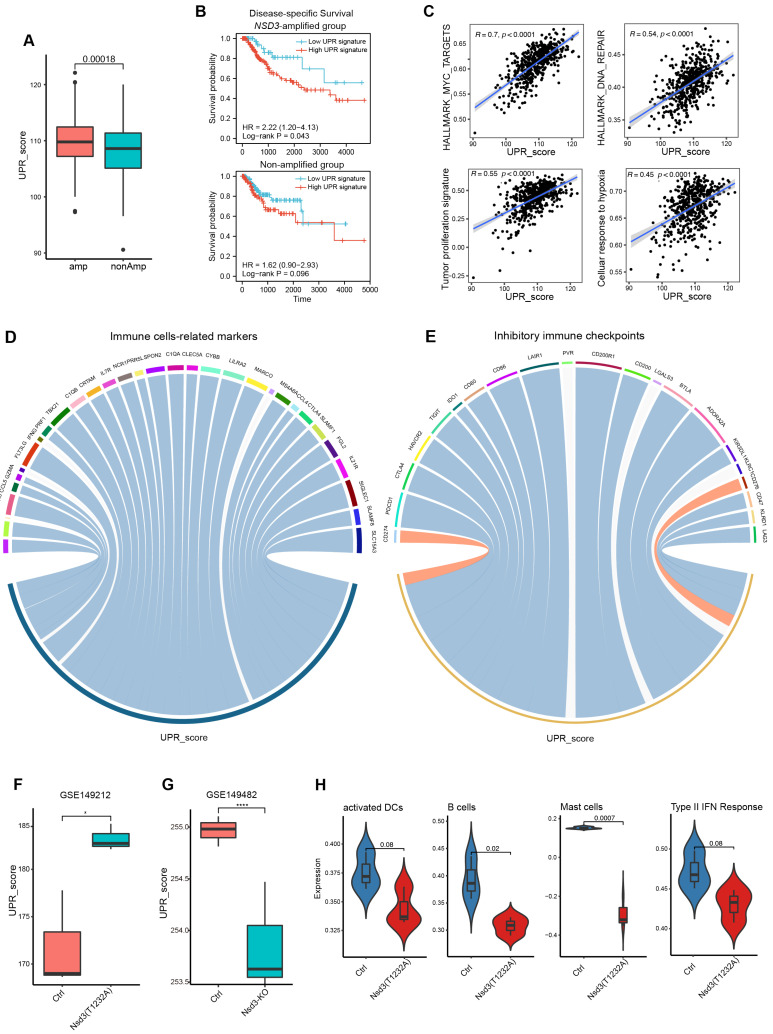
The internal links between UPR and non-immunogenic features of *NSD3*-amplified LUSC. (**A**) The difference in the unfolded protein response (UPR) gene signature between the non- and amplification groups of *NSD3* in the TCGA LUSC cohort (*n* = 491). The UPR gene signature was scored as the sum of the hallmark UPR gene sets. The Wilcoxon rank-sum test was used for comparison, and *p* < 0.05 was considered significant. (**B**) Kaplan–Meier analysis based on the UPR gene score in *NSD3*-amplified LUSC patients (upper panel; *n* = 207) or non-amplified group (lower panel; *n* = 236), with high (in red) or low (in blue) expression, was stratified by the optimal cut-off value of the individual expression across all patients using the surv_cutpoint function in the R “maxstat” package. The log-rank test was used for comparison, and *p* < 0.05 was considered significant. (**C**) Correlation analysis between the UPR gene score and several gene signatures was calculated using the “GSVA” R package in the TCGA LUSC cohort (*n* = 491). (**D**,**E**) Correlation analysis between the UPR gene score and a panel of immune cell-related gene markers (**D**) as well as inhibitory immune checkpoints (**E**) in the TCGA LUSC cohort (*n* = 491). (**F**,**G**) The expression levels of UPR gene signature in the indicated groups. Transcriptomic data were obtained from transgenic mice carrying a lung-specific active mutant (T1232A) or genetic knockout of *Nsd3* (GSE149212 & GSE149482). A two-tailed unpaired *t*-test was used for comparison, and *p* < 0.05 was considered significant. * *p* < 0.05; **** *p* < 0.001. (**H**) The difference in the indicated immune cell types and the type II-IFN response was calculated using the “GSVA” R package between the wild-type and *Nsd3* active mutant (T1232A) groups. A two-tailed unpaired *t*-test was used for comparison, and *p* < 0.05 was considered significant.

Given the enormous enrichment of the UPR in *NSD3*-amplified tumors (Figure 1E) and the tight junction between UPR signaling and anti-tumor immunity [35,36], we sought to determine whether the UPR is associated with the suppressive immunological features of *NSD3*-amplified LUSC. Apart from displaying an upregulated expression pattern (Figure 6A), the high UPR gene signature predicted a poor prognosis, particularly in the *NSD3*-amplified group (Figure 6B, Supplementary Figure S4A). In addition, LUSC carrying high UPR was accompanied by an aggressive phenotype in LUSC, as indicated by the enormously positive correlation with MYC targets, DNA repair, tumor proliferation, and hypoxia signatures (Figure 6C). The above findings indicated that hyperactivated UPR might contribute to the aggressive nature of *NSD3*-amplified LUSC. Of particular interest, the UPR gene signature manifested a significantly negative correlation with immune cell-related gene markers (e.g., SIGLEC1, FLT3LG, SLC15A3, and TBX21) and inhibitory immune checkpoints (e.g., PD-1, CTLA-4, CD47, and CD86) (Figure 6D,E, Supplementary Figure S4A, Tables S10 and S11).

Additionally, we further confirmed that the UPR gene signature was upregulated in the *Nsd3*-mutant group but with a decreased expression pattern in the *Nsd3*-knockout group by mining transcriptomic data (GSE149212 & GSE149482) from transgenic mice carrying a lung-specific active mutant (T1232A) or genetic knockout of *Nsd3* to model the consequences of *NSD3* amplification and depletion (Figure 6F,G, Supplementary Figure S4B,C). Interestingly, ssGSEA (GSVA) showed that the hyperactivity of *NSD3* further accelerated the immune-desert phenotype in the tumor, as indicated by the reduced infiltration of several immune cell types (e.g., activated DCs, B cells, and mast cells) and repressed activities of the type II-IFN response (Figure 6H, Supplementary Figure S4D,E). In brief, we reveal that the UPR might be an essential mediator of the non-inflamed TME phenotype of *NSD3*-amplified LUSC.

### 3.6. Exploration of Potential Therapeutic Targets for NSD3-Amplified LUSC

**Figure 7:**
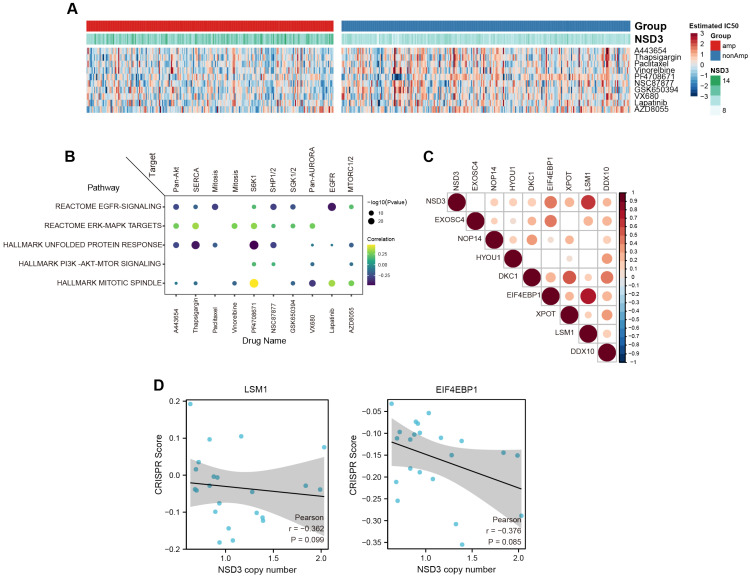
Prioritization of cancer drug targets for *NSD3*-amplified LUSC. (**A**) Heatmap showing the estimated half maximal inhibitory concentration (IC_50_) values of candidate therapeutic drugs calculated using the “pRRophetic” R package across the TCGA LUSC cohort (*n* = 491). The top 10 compounds ranked by the adjusted *p*-value are shown. The Wilcoxon rank-sum test was used for comparison, and *p* < 0.05 was considered significant. (**B**) Correlation analysis between the estimated IC_50_ values of candidate therapeutic drugs and pathway enrichment scores calculated using the “GSVA” R package. (**C**) Heatmap showing the Spearman correlation coefficients between the mRNA levels of *NSD3* and a panel of UPR genes, which displayed an elevated expression pattern in *NSD3*-amplified LUSC. (**D**) Correlation analysis between the copy number of *NSD3* and CRISPR genetic knockout score of EIF4EBP1 and LSM1 across LUSC cell lines (*n* = 22). Data were downloaded from The Cancer Dependency Map Project (DepMap; 22Q2 Chronos).

As clinical NSD3 inhibitors are currently unavailable, we next attempted to explore the potential therapeutic targets for *NSD3*-amplified LUSC by screening 138 clinical chemotherapeutic drugs. The correlation analysis between *NSD3* genetic status and treatment response based on the estimated half maximal inhibitory concentration (IC_50_) revealed that *NSD3*-amplified LUSCs exhibited a more sensitive phenotype to several compounds (e.g., A-443654, thapsigargin, paclitaxel, and lapatinib) than the wild-type group (Figure 7A, Supplementary Table S12). Strikingly, thapsigargin, an endoplasmic reticulum Ca2+ ATPase inhibitor intervening in UPR signaling, was one of the TOP compounds that exhibited promising synthetic lethality with *NSD3* amplification (Figure 7B). In line with this finding, *NSD3* expression was negatively correlated with the IC_50_ of thapsigargin in LUSC cell lines (Supplementary Figure S4F). In addition, we also found that *NSD3* gene expression was tightly associated with several UPR-related genes (e.g., *LSM1*, *EIF4EBP1*, *HYOU1*, and *DDX10*) (Figure 7C). Interestingly, *LSM1* and *EIF4EBP1* were the top candidates among the upregulated DEGs in the *NSD3*-amplified LUSC (Figure 1D, Supplementary Figure S5). More importantly, *NSD3*-amplified LUSC cell lines were accompanied by a more sensitive phenotype to the specific genetic knockout of *LSM1* and *EIF4EBP1* (Figure 7D), which might be candidate synthetic targets for *NSD3*-amplified LUSC. Hence, our findings further indicate that the clinically actionable drugs that targeting UPR signaling might serve as a novel therapeutic rationale for *NSD3*-amplified LUSC.

## 4. Discussion

Tremendous efforts have been made to delineate the molecular underpinnings and immunological profiles of squamous cell lung cancer; however, our knowledge about the TIME of LUSC is still poorly understood. Immune checkpoint blockades alone or combined with standard chemotherapy have achieved considerable success in advanced LUSC patients [37,38]. However, the heterogeneous response and limited predictive biomarkers for immunotherapy are still great challenges in clinical oncology [16,39,40,41]. In the present study, by utilizing multi-scale systematic analysis (e.g., genome, transcriptome, proteome, and TMA array), we provided the first evidence that *NSD3* gene amplification, a newly identified genetic driver of LUSC tumorigenesis, is associated with an immune-desert phenotype and consequently a poor immunotherapy outcome. Further molecular characterizations indicated that the high activity of UPR signaling might mediate the non-immunogenic phenotype of *NSD3*-amplified LUSC. Most importantly, we revealed that intervention of UPR signaling is an actionable therapeutic target for *NSD3*-amplified LUSC, which might also have considerable benefits in overcoming cancer resistance to immunotherapy.

The crucial epigenetic deregulator, *NSD3*, was recently identified as a pivotal mutational driver of LUSC tumorigenesis [10]. However, the immunological role of *NSD3* in driving carcinogenesis has not been systematically studied, especially in large-scale cancer cohorts. Interestingly, according to the molecular basis of LUSC, we found that *NSD3*-amplified tumors were enriched in MYC targets, E2F targets, the G2-M checkpoint, and the unfolded protein response (UPR). Meanwhile, these patients also displayed a decreased expression pattern in various immunological pathways, such as interferon-alpha/beta and the inflammatory response, suggesting that there might be an internal link between *NSD3* and immunological regulation within LUSC. By further integrating multi-scale analyses to systematically evaluate the immunological properties of LUSC, we revealed that *NSD3*-amplified LUSC features consistently downregulated immune-related modulators and reduced infiltration of immune cell signatures of a non-immunogenic phenotype. In parallel, we observed a strong negative correlation between the mRNA expression of *NSD3* and inhibitory immune checkpoints. In line with this, high *NSD3* is associated with a worse immunotherapy outcome, further stressing the possibility of *NSD3* as a potential predictive biomarker for ICBs. Future prospective immunotherapy trials, especially in LUSC, are urgently needed to validate the above findings.

Unfolded protein response signaling has been established as a cell-intrinsic survival mechanism for malignant cells facing microenvironmental stressors [42,43,44], and it could interfere with local anti-tumor immunity [35,36,45,46]. However, its role in LUSC has been insufficiently studied. Apart from an upregulated UPR pattern observed in the *NSD3*-amplified LUSC, our data provided compelling evidence that neither hyperactivity nor depletion of *NSD3* is involved in the regulation of the UPR gene signature. Recent studies have shown that *NSD3*-induced histone methylation is functionally involved in tumor initiation and progression by facilitating the transactivation of oncogenic signaling, such as NOTCH, MYC, and mTORC1 [10,47]. Further studies are warranted to investigate the underlying mechanisms of the *NSD3*-driven UPR response.

In general, tumors can be divided into immune-hot and -cold subtypes based on the levels of immune cell infiltration and the activities of immunomodulators. Increasing evidence demonstrates that the absence of tumor antigens, antigen presentation defects, and T-cell activation contribute to a “cold” tumor-immune microenvironment [48,49]. During the evolution of carcinogenesis, the accumulation of genetic alterations and cancer hallmarks are inherent mechanisms for a “cold tumor” state [50,51,52]. A hyperactivated UPR response has been reported to account for dysfunctional dendritic cells (DCs) and neutrophils [53,54]. Moreover, CHOP, a critical downstream effector of the UPR response, is vital for the immune inhibitory activity of tumor-infiltrating myeloid-derived suppressor cells (MDSCs) and CD8+ T-cells [55]. In line with this, our data revealed that the UPR gene signature manifested a significantly negative correlation with immune cell-related gene markers and inhibitory immune checkpoints, pointing to the possibility that UPR might be a mediator of the “cold tumor” immunological phenotype of LUSC. Therefore, future studies will be interesting to explore how the UPR contributes to the *NSD3*-driven “cold” tumor immune microenvironment.

Until now, immunological treatment of “cold tumors” has been a substantial clinical problem, as no adaptive immune response has been established. Emerging evidence has shown that shifting the anti-tumor immunity to the T-cell inflamed phenotype could be an excellent therapeutic rationale for improving the immunotherapy response [56]. Here, our data showed that the hyperactivity of *NSD3* could further accelerate the immune-desert phenotype; in contrast, the depletion of *NSD3* is accompanied by an upregulated type-II IFN response, suggesting that targeting *NSD3* might be a promising rationale for transforming the TIME. At present, however, there is no *NSD3* inhibitor that can be used in a physiological setting. By integrating pharmaco-transcriptomic analysis, we further proved that the intervention in UPR signaling, especially thapsigargin, might be a novel therapy for *NSD3*-amplified LUSC. Despite the promising efficiency of targeting UPR branches to balance the immune response, investigations of their usefulness in immune modulations, especially in lung cancer, are rare [57]. Notably, a robust/lethal overactivation of the UPR was shown to trigger immunogenic cell death (ICD), which in turn promotes immunostimulatory responses [58,59,60]. In our scenario, we speculate that chemically induced permanent ER stress by thapsigargin could drive tumor cells towards ICD and finally lead to the elevation of the immunogenicity for enhanced cancer therapy. Hence, future studies are warranted to confirm their clinical efficacy, especially as a combination rationale with immunotherapy.

## 5. Limitations

There are some limitations in this study requiring further redress. (1) Albeit our data revealed that both the mRNA and protein levels of *NSD3* might be superior predictors of its amplification state, direct evidence between the genetic status of *NSD3* and the corresponding protein expression is still lacking. (2) A slight inconsistency in the cancer immunity cycle persists between the two applied LUSC patient cohorts, which might be due to the tumor heterogeneity, and the difference in sample size. Validation with independent cohorts is warranted. (3) As expected, *NSD3*-amplified LUSC was associated with a downregulated cytotoxic T-cell signature; however, the internal links between *NSD3* and different subtypes of T-cells (naive, effector, memory, and exhausted T-cells) need to be further elucidated. Multi-colour IHC (mIHC) is highly recommended to dissect the above issue in future studies.

## 6. Conclusions

In summary, we provide the first solid evidence that *NSD3* gene amplification defines a non-immunogenic phenotype in LUSC and is associated with poor immunotherapy outcomes. Further molecular characterization revealed the potential internal links between the unfolded protein response and the “cold” tumor immune microenvironment of *NSD3*-amplified LUSC. More valuable, our data imply that either directly targeting *NSD3* or intervention of UPR signaling might be a promising therapeutic rationale to boost anti-tumor immunity and improve the immunotherapy response.

## Data Availability

The data presented in this study are available in this article (and Supplementary Material).

## Supplementary Materials

The following supporting information can be downloaded at https://www.mdpi.com/article/10.3390/cancers14204997/s1. Figure S1: The genetic landscape of *NSD3*-amplified LUSC; Figure S2. Immunological profiles of *NSD3*-amplified LUSC; Figure S3. The landscape of the LUSC TMA array; Figure S4. The association between UPR and non-immunogenic features of NSD3-amplified LUSC; Figure S5. Overview of the study design; Table S1. DEG analysis based on the amplification state of *NSD3* in the TCGA-LUSC cohort; Table S2. GSEA analysis between non- and amplification groups of *NSD3*; Table S3. The copy number of *NSD3* and CRISPR genetic knockout score across LUSC cell lines; Table S4. Expression pattern of immunomodulators between non- and amplification of *NSD3* in the TCGA-LUSC cohort; Table S5. The infiltration levels of immune cells in LUSC are based on five algorithms (TIMER, EPIC, MCP-counter, quanTIseq, and TISIDB); Table S6. Expression levels of tumor-infiltrating immune cell (TIIC) -related effector genes between the non- and amplification of *NSD3* in TCGA-LUSC cohort; Table S7. Expression levels of immunomodulators between non- and amplification of *NSD3* in CPTAC-LUSC cohort; Table S8. Expression levels of tumor-infiltration immune cells (TIICs) -related effector genes between non- and amplification of *NSD3* in the CPTAC-LUSC cohort; Table S9. Correlation analysis between the expression of *NSD3* and a panel of inhibitory immune checkpoints; Table S10. Correlation analysis between the UPR gene signature and immune cell-related gene markers; Table S11. Correlation analysis between the UPR gene signature and inhibitory immune checkpoints; Table S12. Estimated IC50 values of candidate therapeutic targets for *NSD3*-amplified LUSC.

## References

[1] Sung H., Ferlay J., Siegel R.L., Laversanne M., Soerjomataram I., Jemal A., Bray F. (2021). Global Cancer Statistics 2020: GLOBOCAN Estimates of Incidence and Mortality Worldwide for 36 Cancers in 185 Countries. CA Cancer J. Clin..

[2] Pan Y., Han H., Labbe K.E., Zhang H., Wong K.-K. (2021). Recent advances in preclinical models for lung squamous cell carcinoma. Oncogene.

[3] Kim Y., Hammerman P.S., Kim J., Yoon J.-A., Lee Y., Sun J.-M., Wilkerson M.D., Pedamallu C.S., Cibulskis K., Yoo Y.K. (2014). Integrative and Comparative Genomic Analysis of Lung Squamous Cell Carcinomas in East Asian Patients. J. Clin. Oncol..

[4] Campbell J.D., Alexandrov A., Kim J., Wala J., Berger A.H., Pedamallu C.S., Shukla S.A., Guo G., Brooks A.N., Murray B.A. (2016). Distinct patterns of somatic genome alterations in lung adenocarcinomas and squamous cell carcinomas. Nat. Genet..

[5] Paik P.K., Pillai R.N., Lathan C.S., Velasco S.A., Papadimitrakopoulou V. (2019). New Treatment Options in Advanced Squamous Cell Lung Cancer. Am. Soc. Clin. Oncol. Educ. Book.

[6] Weiss J., Sos M.L., Seidel D., Peifer M., Zander T., Heuckmann J.M., Ullrich R.T., Menon R., Maier S., Soltermann A. (2010). Frequent and Focal *FGFR1* Amplification Associates with Therapeutically Tractable FGFR1 Dependency in Squamous Cell Lung Cancer. Sci. Transl. Med..

[7] Rooney C., Geh C., Williams V., Heuckmann J.M., Menon R., Schneider P., Al-Kadhimi K., Dymond M., Smith N.R., Baker D. (2016). Characterization of FGFR1 Locus in sqNSCLC Reveals a Broad and Heterogeneous Amplicon. PLoS ONE.

[8] Babina I.S., Turner N.C. (2017). Advances and challenges in targeting FGFR signalling in cancer. Nat. Rev. Cancer.

[9] Paik P.K., Shen R., Berger M.F., Ferry D., Soria J.-C., Mathewson A., Rooney C., Smith N.R., Cullberg M., Kilgour E. (2017). A Phase Ib Open-Label Multicenter Study of AZD4547 in Patients with Advanced Squamous Cell Lung Cancers. Clin. Cancer Res..

[10] Yuan G., Flores N.M., Hausmann S., Lofgren S.M., Kharchenko V., Angulo-Ibanez M., Sengupta D., Lu X., Czaban I., Azhibek D. (2021). Elevated NSD3 histone methylation activity drives squamous cell lung cancer. Nature.

[11] Turner-Ivey B., Smith E.L., Rutkovsky A.C., Spruill L.S., Mills J.N., Ethier S.P. (2017). Development of mammary hyperplasia, dysplasia, and invasive ductal carcinoma in transgenic mice expressing the 8p11 amplicon oncogene NSD3. Breast Cancer Res. Treat..

[12] Binnewies M., Roberts E.W., Kersten K., Chan V., Fearon D.F., Merad M., Coussens L.M., Gabrilovich D.I., Ostrand-Rosenberg S., Hedrick C.C. (2018). Understanding the tumor immune microenvironment (TIME) for effective therapy. Nat. Med..

[13] Jenkins R.W., Barbie D.A., Flaherty K.T. (2018). Mechanisms of resistance to immune checkpoint inhibitors. Br. J. Cancer.

[14] Fares C.M., Van Allen E.M., Drake C.G., Allison J.P., Hu-Lieskovan S. (2019). Mechanisms of Resistance to Immune Checkpoint Blockade: Why Does Checkpoint Inhibitor Immunotherapy Not Work for All Patients?. Am. Soc. Clin. Oncol. Educ. Book.

[15] Shembrey C., Huntington N.D., Hollande F. (2019). Impact of Tumor and Immunological Heterogeneity on the Anti-Cancer Immune Response. Cancers.

[16] Li R., Lin Y., Wang Y., Wang S., Yang Y., Mu X., Chen Y., Gao Z. (2021). Characterization of the Tumor Immune Microenvironment in Lung Squamous Cell Carcinoma Using Imaging Mass Cytometry. Front. Oncol..

[17] Wang C., Wang Q., Xu X., Xie B., Zhao Y., Li N., Cao X. (2017). The methyltransferase NSD3 promotes antiviral innate immunity via direct lysine methylation of IRF3. J. Exp. Med..

[18] Satpathy S., Krug K., Beltran P.M.J., Savage S.R., Petralia F., Kumar-Sinha C., Dou Y., Reva B., Kane M.H., Avanessian S.C. (2021). A proteogenomic portrait of lung squamous cell carcinoma. Cell.

[19] Jung H., Kim H.S., Kim J.Y., Sun J.-M., Ahn J.S., Ahn M.-J., Park K., Esteller M., Lee S.-H., Choi J.K. (2019). DNA methylation loss promotes immune evasion of tumours with high mutation and copy number load. Nat. Commun..

[20] Tsherniak A., Vazquez F., Montgomery P.G., Weir B.A., Kryukov G., Cowley G.S., Gill S., Harrington W.F., Pantel S., Krill-Burger J.M. (2017). Defining a Cancer Dependency Map. Cell.

[21] Yang W., Soares J., Greninger P., Edelman E.J., Lightfoot H., Forbes S., Bindal N., Beare D., Smith J.A., Thompson I.R. (2013). Genomics of Drug Sensitivity in Cancer (GDSC): A resource for therapeutic biomarker discovery in cancer cells. Nucleic Acids Res..

[22] Yoshihara K., Shahmoradgoli M., Martínez E., Vegesna R., Kim H., Torres-Garcia W., Trevino V., Shen H., Laird P.W., Levine D.A. (2013). Inferring tumour purity and stromal and immune cell admixture from expression data. Nat. Commun..

[23] Charoentong P., Finotello F., Angelova M., Mayer C., Efremova M., Rieder D., Hackl H., Trajanoski Z. (2017). Pan-cancer Immunogenomic Analyses Reveal Genotype-Immunophenotype Relationships and Predictors of Response to Checkpoint Blockade. Cell Rep..

[24] Hu J., Yu A., Othmane B., Qiu D., Li H., Li C., Liu P., Ren W., Chen M., Gong G. (2021). Siglec15 shapes a non-inflamed tumor microenvironment and predicts the molecular subtype in bladder cancer. Theranostics.

[25] Li T., Fu J., Zeng Z., Cohen D., Li J., Chen Q., Li B., Liu X.S. (2020). TIMER2.0 for analysis of tumor-infiltrating immune cells. Nucleic Acids Res..

[26] Racle J., De Jonge K., Baumgaertner P., Speiser D.E., Gfeller D. (2017). Simultaneous enumeration of cancer and immune cell types from bulk tumor gene expression data. eLife.

[27] Becht E., Giraldo N.A., Lacroix L., Buttard B., Elarouci N., Petitprez F., Selves J., Laurent-Puig P., Sautes-Fridman C., Fridman W.H. (2016). Estimating the population abundance of tissue-infiltrating immune and stromal cell populations using gene expression. Genome Biol..

[28] Finotello F., Mayer C., Plattner C., Laschober G., Rieder D., Hackl H., Krogsdam A., Loncova Z., Posch W., Wilflingseder D. (2019). Molecular and pharmacological modulators of the tumor immune contexture revealed by deconvolution of RNA-seq data. Genome Med..

[29] Ru B., Wong C.N., Tong Y., Zhong J.Y., Zhong S.S.W., Wu W.C., Chu K.C., Wong C.Y., Lau C.Y., Chen I. (2019). TISIDB: An integrated repository portal for tumor-immune system interactions. Bioinformatics.

[30] Bindea G., Mlecnik B., Tosolini M., Kirilovsky A., Waldner M., Obenauf A.C., Angell H., Fredriksen T., Lafontaine L., Berger A. (2013). Spatiotemporal Dynamics of Intratumoral Immune Cells Reveal the Immune Landscape in Human Cancer. Immunity.

[31] Xu L., Deng C., Pang B., Zhang X., Liu W., Liao G., Yuan H., Cheng P., Li F., Long Z. (2018). TIP: A Web Server for Resolving Tumor Immunophenotype Profiling. Cancer Res..

[32] Auslander N., Zhang G., Lee J.S., Frederick D.T., Miao B., Moll T., Tian T., Wei Z., Madan S., Sullivan R.J. (2018). Robust prediction of response to immune checkpoint blockade therapy in metastatic melanoma. Nat. Med..

[33] Ayers M., Lunceford J., Nebozhyn M., Murphy E., Loboda A., Kaufman D.R., Albright A., Cheng J.D., Kang S.P., Shankaran V. (2017). IFN-gamma-related mRNA profile predicts clinical response to PD-1 blockade. J. Clin. Investig..

[34] Geeleher P., Cox N., Huang R.S. (2014). pRRophetic: An R Package for Prediction of Clinical Chemotherapeutic Response from Tumor Gene Expression Levels. PLoS ONE.

[35] Grootjans J., Kaser A., Kaufman R.J., Blumberg R.S. (2016). The unfolded protein response in immunity and inflammation. Nat. Rev. Immunol..

[36] Zanetti M., Xian S., Dosset M., Carter H. (2022). The Unfolded Protein Response at the Tumor-Immune Interface. Front. Immunol..

[37] Mok T.S.K., Wu Y.-L., Kudaba I., Kowalski D.M., Cho B.C., Turna H.Z., Castro G., Srimuninnimit V., Laktionov K.K., Bondarenko I. (2019). Pembrolizumab versus chemotherapy for previously untreated, PD-L1-expressing, locally advanced or metastatic non-small-cell lung cancer (KEYNOTE-042): A randomised, open-label, controlled, phase 3 trial. Lancet.

[38] Paz-Ares L., Ciuleanu T.-E., Cobo M., Schenker M., Zurawski B., Menezes J., Richardet E., Bennouna J., Felip E., Juan-Vidal O. (2021). First-line nivolumab plus ipilimumab combined with two cycles of chemotherapy in patients with non-small-cell lung cancer (CheckMate 9LA): An international, randomised, open-label, phase 3 trial. Lancet Oncol..

[39] Duffy M.J., Crown J. (2019). Biomarkers for Predicting Response to Immunotherapy with Immune Checkpoint Inhibitors in Cancer Patients. Clin. Chem..

[40] Borcoman E., Nandikolla A., Long G., Goel S., Le Tourneau C. (2018). Patterns of Response and Progression to Immunotherapy. Am. Soc. Clin. Oncol. Educ. Book.

[41] Vitale I., Shema E., Loi S., Galluzzi L. (2021). Intratumoral heterogeneity in cancer progression and response to immunotherapy. Nat. Med..

[42] Xu D., Liang S.-Q., Yang H., Lüthi U., Riether C., Berezowska S., Marti T.M., Hall S.R.R., Bruggmann R., Kocher G.J. (2018). Increased sensitivity to apoptosis upon endoplasmic reticulum stress-induced activation of the unfolded protein response in chemotherapy-resistant malignant pleural mesothelioma. Br. J. Cancer.

[43] Cerezo M., Lehraiki A., Millet A., Rouaud F., Plaisant M., Jaune E., Botton T., Ronco C., Abbe P., Amdouni H. (2016). Compounds Triggering ER Stress Exert Anti-Melanoma Effects and Overcome BRAF Inhibitor Resistance. Cancer Cell.

[44] Xu D., Yang H., Berezowska S., Gao Y., Liang S.-Q., Marti T.M., Hall S.R.R., Dorn P., Kocher G.J., Schmid R.A. (2019). Endoplasmic Reticulum Stress Signaling as a Therapeutic Target in Malignant Pleural Mesothelioma. Cancers.

[45] Walter P., Ron D. (2011). The Unfolded Protein Response: From Stress Pathway to Homeostatic Regulation. Science.

[46] Hetz C., Chevet E., Oakes S.A. (2015). Proteostasis control by the unfolded protein response. Nat. Cell Biol..

[47] Jeong G.-Y., Park M.K., Choi H.-J., An H.W., Park Y.-U., Choi H.-J., Park J., Kim H.-Y., Son T., Lee H. (2021). NSD3-Induced Methylation of H3K36 Activates NOTCH Signaling to Drive Breast Tumor Initiation and Metastatic Progression. Cancer Res..

[48] Sharma P., Hu-Lieskovan S., Wargo J.A., Ribas A. (2017). Primary, Adaptive, and Acquired Resistance to Cancer Immunotherapy. Cell.

[49] Bonaventura P., Shekarian T., Alcazer V., Valladeau-Guilemond J., Valsesia-Wittmann S., Amigorena S., Caux C., Depil S. (2019). Cold Tumors: A Therapeutic Challenge for Immunotherapy. Front. Immunol..

[50] Spranger S., Bao R., Gajewski T.F. (2015). Melanoma-intrinsic beta-catenin signalling prevents anti-tumour immunity. Nature.

[51] Peng W., Chen J.Q., Liu C., Malu S., Creasy C., Tetzlaff M.T., Xu C., McKenzie J.A., Zhang C., Liang X. (2016). Loss of PTEN Promotes Resistance to T Cell–Mediated Immunotherapy. Cancer Discov..

[52] Tauriello D.V.F., Palomo-Ponce S., Stork D., Berenguer-Llergo A., Badia-Ramentol J., Iglesias M., Sevillano M., Ibiza S., Canellas A., Hernando-Momblona X. (2018). TGFbeta drives immune evasion in genetically reconstituted colon cancer metastasis. Nature.

[53] Cubillos-Ruiz J.R., Silberman P.C., Rutkowski M.R., Chopra S., Perales-Puchalt A., Song M., Zhang S., Bettigole S.E., Gupta D., Holcomb K. (2015). ER Stress Sensor XBP1 Controls Anti-tumor Immunity by Disrupting Dendritic Cell Homeostasis. Cell.

[54] Condamine T., Kumar V., Ramachandran I.R., Youn J.-I., Celis E., Finnberg N., El-Deiry W., Winograd R., Vonderheide R.H., English N.R. (2014). ER stress regulates myeloid-derived suppressor cell fate through TRAIL-R–mediated apoptosis. J. Clin. Investig..

[55] Thevenot P.T., Sierra R.A., Raber P.L., Al-Khami A.A., Trillo-Tinoco J., Zarreii P., Ochoa A.C., Cui Y., Del Valle L., Rodriguez P.C. (2014). The Stress-Response Sensor Chop Regulates the Function and Accumulation of Myeloid-Derived Suppressor Cells in Tumors. Immunity.

[56] Galon J., Bruni D. (2019). Approaches to treat immune hot, altered and cold tumours with combination immunotherapies. Nat. Rev. Drug Discov..

[57] Li A., Song N.-J., Riesenberg B.P., Li Z. (2019). The Emerging Roles of Endoplasmic Reticulum Stress in Balancing Immunity and Tolerance in Health and Diseases: Mechanisms and Opportunities. Front. Immunol..

[58] Pol J., Vacchelli E., Aranda F., Castoldi F., Eggermont A., Cremer I., Sautes-Fridman C., Fucikova J., Galon J., Spisek R. (2015). Trial Watch: Immunogenic cell death inducers for anticancer chemotherapy. OncoImmunology.

[59] Mohamed E., Cao Y., Rodriguez P.C. (2017). Endoplasmic reticulum stress regulates tumor growth and anti-tumor immunity: A promising opportunity for cancer immunotherapy. Cancer Immunol. Immunother..

[60] Chen X., Cubillos-Ruiz J.R. (2021). Endoplasmic reticulum stress signals in the tumour and its microenvironment. Nat. Rev. Cancer.

