# Supplementary material for: Dissecting the Immunological Profiles in NSD3-Amplified LUSC through Integrative Multi-Scale Analyses
Source: Cancers (Basel). 2022 Oct 12;14(20):4997. doi: 10.3390/cancers14204997 (PMC9599511; doi:10.3390/cancers14204997)
Supplement: Supplementary file 1 [file cancers-14-04997-s001.zip › cancers-1921203-supplementary_2022.09.23.pdf]

Supplementary Materials: Dissecting the immunological profiles in *NSD3*-amplified LUSC through integrative multi-scale analyses

Duo Xu, Shengchen Liu, Xi Wu, Thomas M. Marti, Patrick Dorn, Ralph A. Schmid, Ren-Wang Peng and Yongqian Shu

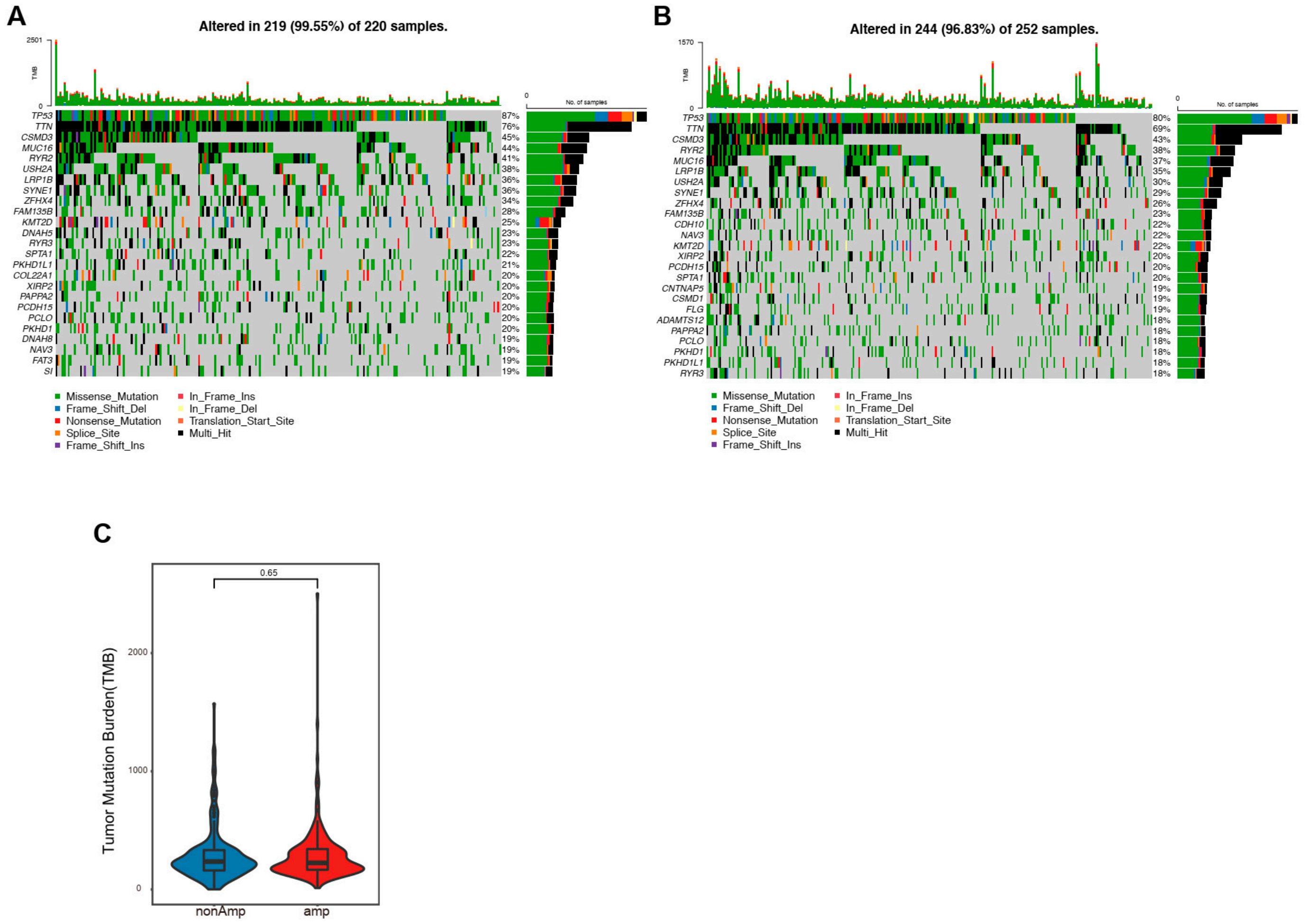

Figure S1. The genetic landscape of *NSD3*-amplified LUSC.

A-B, The genomic mutational profiles in the non- (B) and amplification groups (A) of *NSD3* across the TCGA LUSC cohort (n=491). The top 25 mainly altered genes are shown. **C,** The difference in tumor mutation burden (TMB) between the non- and amplification groups of *NSD3* across the TCGA LUSC cohort (n=491). Wilcoxon rank-sum test was used for comparison, and p<0.05 was considered significant.

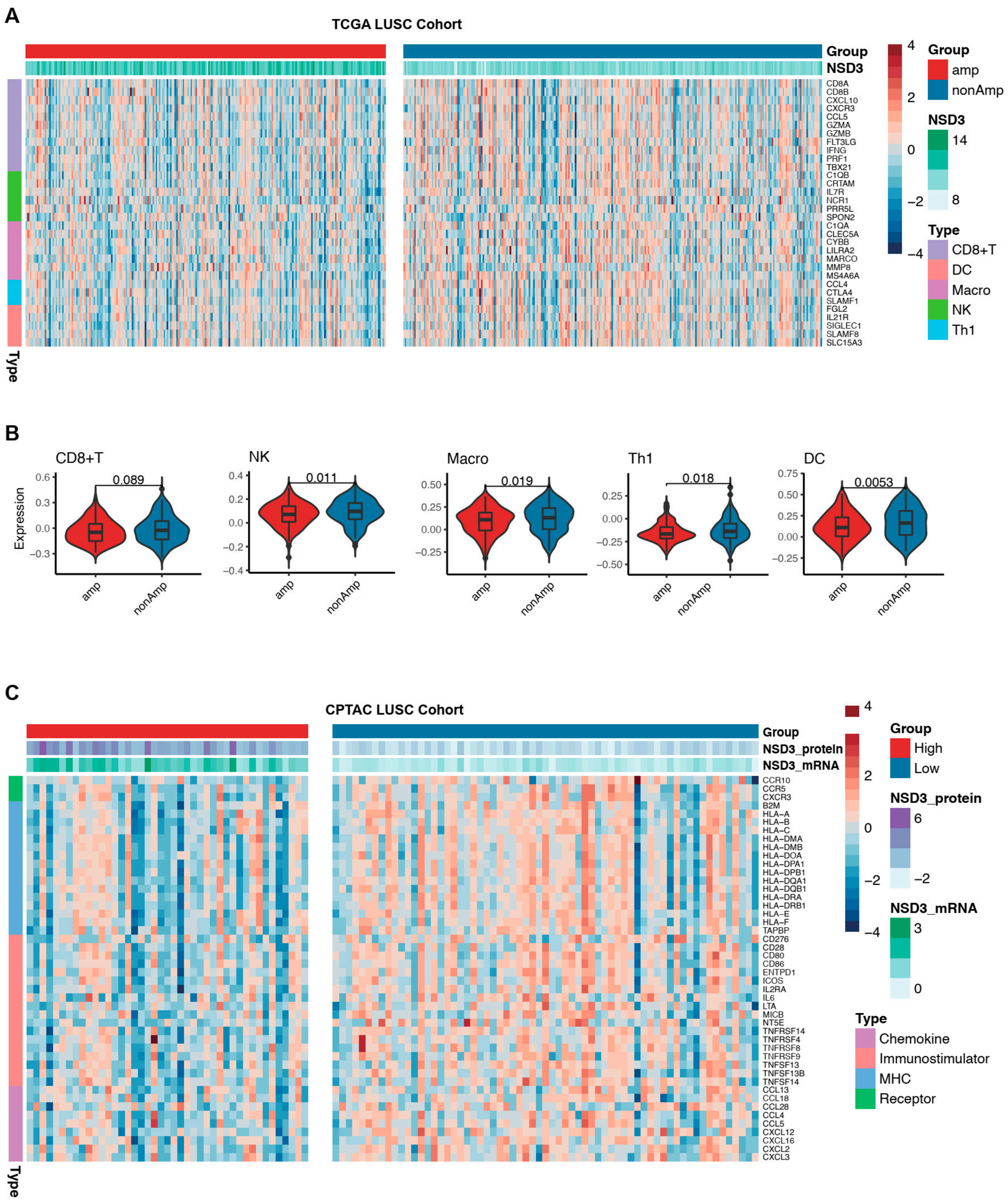

Figure S2. Immunological profiles of *NSD3*-amplified LUSC.

A, Heatmap showing the mRNA expression of immune cell-related genes in the non- and amplification groups of *NSD3* across the TCGA LUSC cohort (n=491). **B,** The difference in immune cell infiltration based on GSVA analysis between the non- and amplification groups of *NSD3* across the TCGA LUSC cohort (n=491). Wilcoxon rank-sum test was used for comparison, and $p<0.05$ was considered significant. **C,** The expression levels of immunomodulators (chemokines, immunostimulators, MHC, and receptors) in the NSD3-low and NSD3-high groups according to the corresponding protein

levels at the cut-off of zero. Data were downloaded from the CPTAC LUSC cohort (n=108). Wilcoxon rank-sum test was used for comparison, and $p<0.05$ was considered significant. Data with statistical significance are shown.

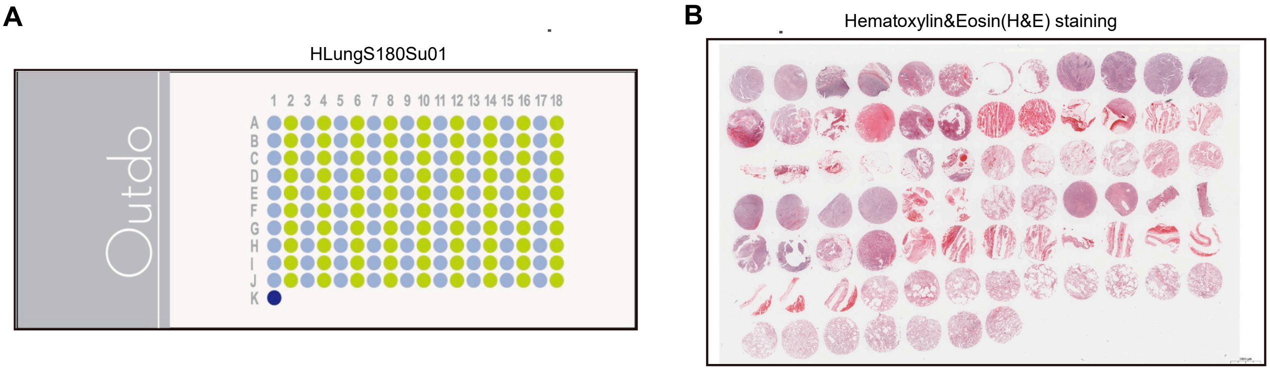

Figure S3. The landscape of the LUSC TMA array.

A-B, Distribution of tissue samples in LUSC TMA array (A). blue dots: tumor samples(n=90); yellow dots: para-tumor (n=90). Representative hematoxylin Eosin (H&E) staining image is shown (B).

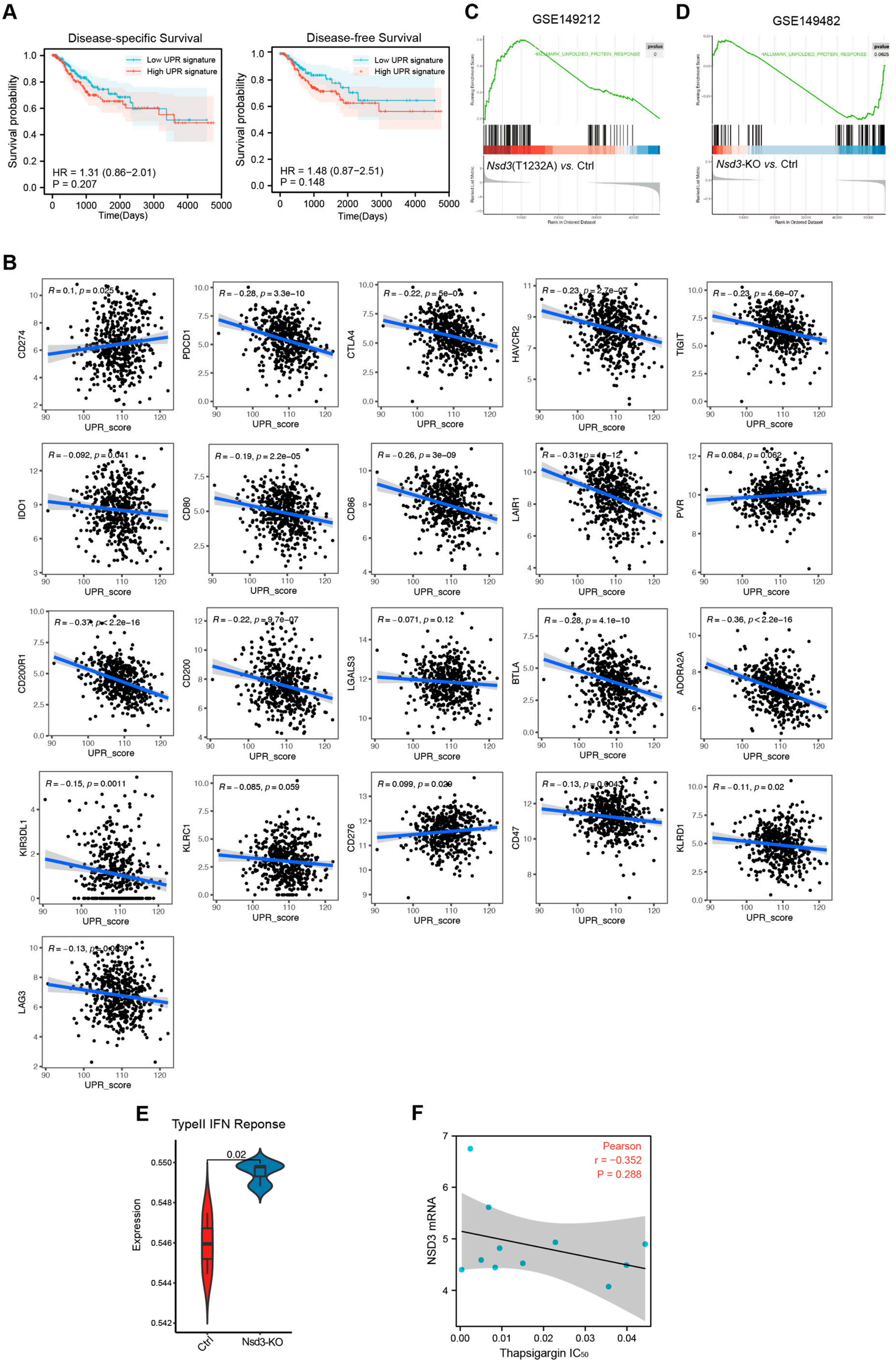

Figure S4. The association between the UPR and non-immunogenic features of *NSD3*-amplified LUSC.

A, Kaplan–Meier analysis based on the UPR gene score in LUSC patients (n=491) with high (in red) or low (in blue) expression was stratified by the optimal cut-off value of the individual expression across all patients using the surv_cutpoint function in the R "maxstat" package. Log-rank test was used for comparison, and $p<0.05$ was considered significant. **B,** Correlation analysis between the UPR gene signature and a panel of the inhibitory immune checkpoints in TCGA LUSC cohort (n=491). **C-D,** GSEA analysis of the UPR gene signature. Transcriptomic data were obtained from transgenic mice carrying a lung-specific active mutant (T1232A) or genetic knockout of *Nsd3* (GSE149212&GSE149482) **E,** Activities of type II-IFN response calculated using the "GSVA" R package in wild-type and *Nsd3*-knockout groups. A two-tailed unpaired t-test was used for comparison, and $p<0.05$ was considered significant. Transcriptomic data were obtained from GSE149482. **(F)** Correlation analysis between the mRNA expression of NSD3 and IC_{50} of Thapsigargin across LUSC cell lines (n=11). Data were downloaded from Genomics of Drug Sensitivity in Cancer (GDSC).

Table S1. DEGs analysis based on the amplification state of *NSD3* in the TCGA-LUSC cohort.

Table S2. GSEA analysis between non- and amplification groups of *NSD3*.

Table S3. The copy number of *NSD3* and CRISPR genetic knockout score across LUSC cell lines.

Table S4. Expression pattern of immunomodulators between non- and amplification of *NSD3* in TCGA-LUSC cohort.

Table S5. The infiltration levels of immune cells in LUSC are based on five algorithms (TIMER, EPIC, MCP-counter, quanTIseq, and TISIDB).

Table S6. Expression levels of tumor-infiltration immune cells (TIICs) -related effector genes between non- and amplification of *NSD3* in TCGA-LUSC cohort.

Table S7. Expression levels of immunomodulators between non- and amplification of *NSD3* in CPTAC-LUSC cohort.

Table S8. Expression levels of tumor-infiltration immune cells (TIICs) -related effector genes between non- and amplification of *NSD3* in CPTAC-LUSC cohort.

Table S9. Correlation analysis between the expression of *NSD3* and a panel of inhibitory immune checkpoints.

Table S10. Correlation analysis between UPR gene signature and immune cell-related gene markers.

Table S11. Correlation analysis between UPR gene signature and inhibitory immune checkpoints.

Table S12. Estimated IC_{50} values of candidate therapeutic targets for *NSD3*-amplified LUSC.
